# Nitazoxanide and quercetin co-loaded nanotransfersomal gel for topical treatment of cutaneous leishmaniasis with macrophage targeting and enhanced anti-leishmanial effect

**DOI:** 10.1016/j.heliyon.2023.e21939

**Published:** 2023-11-02

**Authors:** Sidra Bashir, Kanwal Shabbir, Fakhar ud Din, Saif Ullah Khan, Zakir Ali, Barkat Ali Khan, Dong Wuk Kim, Gul Majid Khan

**Affiliations:** a Nanomedicine Research Group, Department of Pharmacy, Faculty of Biological Sciences, Quaid-i-Azam University Islamabad, Pakistan; b Department of Pharmacy, Faculty of Biological Sciences, Quaid-i-Azam University Islamabad, Pakistan; c Institute of Biotechnology and Microbiology, Bacha Khan University, Charsada, KPK, Pakistan; d Drugs Design and Cosmetics Lab (DDCL), Faculty of Pharmacy Gomal University, Dera Ismail Khan, Pakistan; e College of Pharmacy, Research Institute of Pharmaceutical Sciences, Kyungpook National University, Daegu, 41566, South Korea; f Islamia College University, Peshawar, Khyber Pakhtunkhwa, Pakistan

**Keywords:** Cutaneous leishmaniasis, Nitazoxanide, Quercetin, Transfersomes, Macrophage targeting

## Abstract

**Purpose:**

Anti-leishmanial medications administered by oral and parenteral routes are less effective for treatment of cutaneous leishmaniasis (CL) and cause toxicity, hence targeted drug delivery is an efficient way to improve drug availability for CL with reduced toxicity. This study aimed to develop, characterize and evaluate nitazoxanide and quercetin co-loaded nanotransfersomal gel (NTZ-QUR-NTG) for the treatment of CL.

**Methods:**

NTZ-QUR-NT were prepared by thin film hydration method and were statistically optimized using Box-Behnken design. To ease the topical delivery and enhance the retention time, the NTZ-QUR-NT were dispersed in 2 % chitosan gel. Moreover, in-vitro drug release, ex-vivo permeation, macrophage uptake, cytotoxicity and anti-leishmanial assays were performed.

**Results:**

The optimized formulation indicated mean particle size 210 nm, poly dispersity index (PDI) 0.16, zeta potential (ZP) −15.1 mV and entrapment efficiency (EE) of NTZ and QUR was 88 % and 85 %, respectively. NTZ-QUR-NT and NTZ-QUR-NTG showed sustained release of the incorporated drugs as compared to the drug dispersions. Skin permeation of NTZ and QUR in NTZ-QUR-NTG was 4 times higher in comparison to the plain gels. The NTZ-QUR-NT cell internalization was almost 10-folds higher than NTZ-QUR dispersion. The cytotoxicity potential (CC_50_) of NTZ-QUR-NT (71.95 ± 3.32 μg/mL) was reduced as compared to NTZ-QUR dispersion (49.77 ± 2.15 μg/mL. A synergistic interaction was found between NTZ and QUR. Moreover, in-vitro anti-leishmanial assay presented a lower IC_50_ value of NTZ-QUR-NT as compared to NTZ-QUR dispersion. Additionally, a significantly reduced lesion size was observed in NTZ-QUR-NTG treated BALB/c mice, indicating its antileishmanial potential.

**Conclusion:**

It can be concluded that nanotransfersomal gel has the capability to retain and permeate the incorporated drugs through stratum corneum and induce synergetic anti-leishmanial effect of NTZ and QUR against cutaneous leishmaniasis.

## 1 Introduction

*Leishmaniasis* is a parasitic infection which is common in tropical areas and being neglected for decades, despite being the ninth major cause of diseases among globally infected individuals [1,2]. It is caused by intracellular species of kinetoplastid protozoan from genus *Leishmania* and transmitted by vector sand fly of genus *Phlebotomus and Lutzomiya*. It is considered to be a disease of poor people living in developing countries and having low socioeconomic status [3]. Across the globe, 98 nations are endemic with leishmaniasis and over 600 million individuals are at risk of visceral *Leishmania* and 1 million at risk of cutaneous *Leishmania* per year [4]. *Leishmaniasis* can be classified into two main types visceral *Leishmaniasis* and tegumentary *Leishmaniasis*. The tegumentary *Leishmaniasis* is further classified on basis of clinical manifestation into cutaneous, mucosal and mucocutaneous *Leishmaniasis* [5,6]. Among them, cutaneous *Leishmaniasis* (CL) is the most prevalent form which causes a variety of symptoms on the skin, including tiny nodules, plaques, and ulcerative lesions [7]. Lack of effective CL vaccinations and limited therapeutic alternatives highlighted the need for research into novel therapeutics from both the natural and synthetic drug pools. Numerous authors have emphasized on the need of considering drug combinations in anti-leishmanial therapy as the combination therapy is widely used for other infectious ailments such as, AIDS, malaria and cancer [8,9]. The combination therapy is thought to be the most successful and advantageous because it outperforms monotherapy even at much lower doses [10,11].

**Fig. 1:**
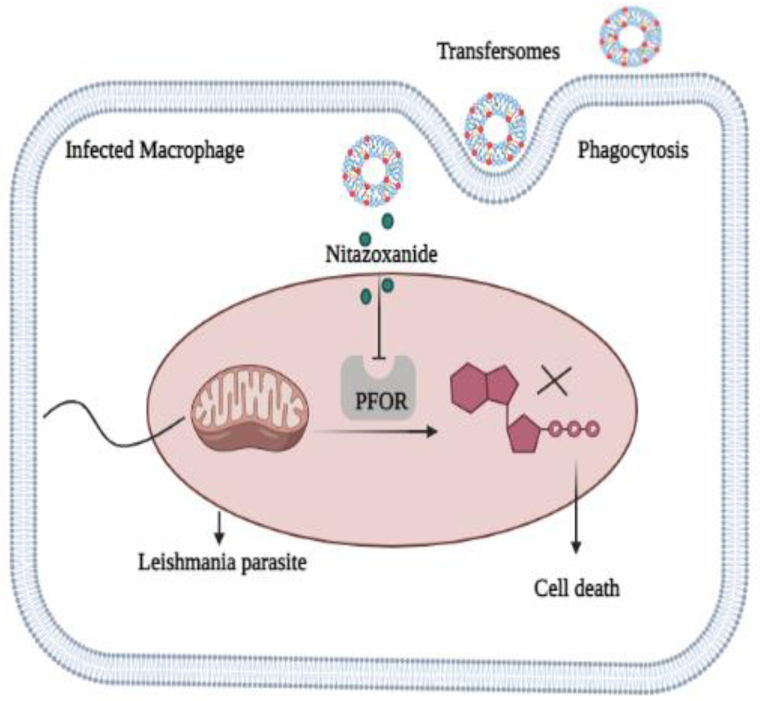
Nitazoxanide exhibits its anti-leishmanial effect by inhibiting the pyruvate ferredoxin oxidoreductase (PFOR) enzyme which leads to decrease in ATP and cause Leishmania death. (Figure is created via Biorender).

**Fig. 2:**
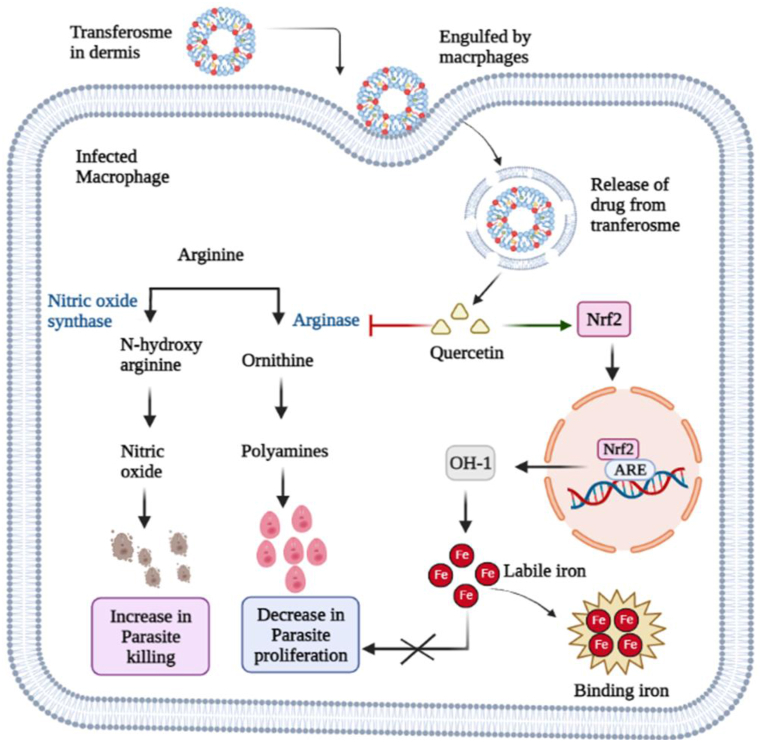
Anti-leishmanial effect of Quercetin is explained by two mechanisms. First, it inhibits the enzyme arginase which leishmania parasites use for their polyamines production. Secondly, it activates Nrf2 regulator of ARF motif genes which cause chelation via OH-1 and reduce labile iron, impeding the leishmania iron dependent replication inside the macrophage (Figure is created via Biorender).

Currently, anti-parasitic drugs that cause oxidative stress like increased production of reactive oxygen species and inhibit redox enzyme are considered novel therapeutic candidate for the treatment of *Leishmaniasis* [12]. Nitazoxanide [2-(5-nitrothiazol-2-ylcarbamoyl) phenyl acetate] is a new anti-parasitic agent belongs to a nitro heterocyclic class named thiazolide [13,14]. Nitazoxanide (NTZ) shows its anti-parasitic action by inhibiting the enzyme pyruvate ferredoxin oxidoreductase (PFOR) which inhibits the ATP production and eventually cause parasites death as illustrated in Fig. 1. In comparison to other alternative drugs, NTZ is more selective against parasites than bacterial or fungal infections and has the potential to avoid resistance mechanisms [14,15]. Quercetin (QUR) is a natural compound and most commonly used flavonoid because of its anti-cancer, anti-inflammatory, anti-oxidation and anti-leishmanial properties [16]. QUR shows its anti-leishmanial effect by inhibiting the arginase, an important enzyme for parasite proliferation as explained in Fig. 2. Another study showed that it is potent activator of nuclear factor-erythroid 2 related factor 2 (Nrf2) which plays very important role for the regulation of genes that carry ARE motif like ferroportin and H-ferritin that are involved in regulation of labile iron. The labile iron is used by *Leishmania* parasite inside the macrophage for replication and survival. But, QUR acts as iron chelator by using Nrf2/OH-1 to decrease the concentration of labile iron and increase the bound iron. As no labile iron will be available for parasite which may leads to its death [17,18]. QUR has been proposed as potential candidate in combination with other anti-leishmanial agents for the treatment of cutaneous *Leishmaniasis* [19]. Due to the anti-parasitic properties of NTZ and QUR and a need to produce new treatment for *Leishmaniasis*, the synergistic effects of NTZ and QUR in combination with each other was investigated in this study.

Parenteral treatment for VL is effective in lowering parasite levels in the spleen and liver, but it has serious adverse effects and complications when used to treat CL [2]. Moreover, systemic medication delivery in CL is associated with considerable toxicity, requires careful monitoring, and nevertheless yields undesired outcomes [20]. Topical drug delivery system is considered as best route by world health organization (WHO) for the treatment of cutaneous *Leishmaniasis* owing to its advantages over oral and parenteral routes [21]. Topical route is preferred because it provides extended drug release, localized action without first pass metabolism and systemic toxicity [22]. The major concern in topical delivery is penetration through stratum corneum (SC) to reach the deeper skin layers, where the desired action is required [23,24]. Therefore, an ultimate solution to this problem is to develop a delivery system which can overcome the upper skin layers barrier, enter the macrophages and release the drug [25,26]. Such a system can be developed with nanotransfersomes (NT) for efficient therapy, which because of their flexibility squeeze through the tight junction of SC and facilitate the penetration of drugs [27]. The aim of the current study was to develop NT loaded with NTZ-QUR for their synergistic effect and passively target the *Leishmania* parasites residing in macrophages of the dermal layer of skin. Additionally, the NT were incorporated into chitosan gel to ensure their retention at the target site. Extensive characterization of the developed formulation was performed including their optimization, in vitro and ex vivo evaluation and cytotoxicity assay.

## 2 Materials and methods

### 2.1 Materials

Quercetin was purchased from Sigma Aldrich (Hamburg, Germany) and nitazoxanide was obtained from Macklin Biochemical Co., Ltd (Shanghai, China). Chitosan, Tween® 80, methanol and chloroform were procured from Sigma Aldrich (Tokyo, Japan). Phospholipon 90G (PL90G) was gifted by lipoid AG (Steinhausen, Switzerland). Roswell Park Memorial Institute (RPMI-1640) media and penicillin/streptomycin were purchased from Thermo Fisher Scientific, Carlsbad, CA USA. Dialysis membranes (12–14 kDa) were procured from Medicell Membranes Ltd, (London, UK). All other chemicals used in this study were of pure analytical grade.

### 2.2 Animals and parasites

Male Albino Wister rats of weight 100–120 g, and BALB/c mice 35–40 g were purchased from National Institute of Health (NIH), Islamabad. The animals were housed in standard facilities in accordance with the NIH recommendations for the care and management of laboratory animals. Moreover, ARRIVE guidelines (II) were followed for animal study. Bioethical approval for animal study was obtained from the bioethical committee of Quaid-i-Azam University, Islamabad, Pakistan (Protocol no BEC-FBS-QAU2021-364). *L*. *tropica* were procured from Khyber Medical University Peshawar KPK, Pakistan**.**

### 2.3 Methods

#### 2.3.1 Preparation of NTZ-QUR-NT

NTZ-QUR-NT were prepared by employing thin film hydration method (as illustrated in Supplemental Fig. S1). Initially, Phospholipon 90G (PL90G), surfactant (Tween® 80), NTZ and QUR (concentrations are mentioned in Table 1) were added in methanol (5 mL) and chloroform (5 mL) mixture to prepare organic phase. Then, the organic phase was transferred to a rotary evaporator flask and subjected to reduced pressure at 50 °C. The thin film obtained by organic phase evaporation was hydrated with phosphate buffer saline (PBS) for 1 h at 60 °C. Finally, the formulation was sonicated for 5 min and to get homogenized NT extrusion was done through 0.45 μm and 0.22 μm filter [28,29].

**Table 1:** Optimization table of the NTZ-QUR-NT using Design Expert® via Box-Behnke design.

Run	Lipid (mg)	Surfactant (mg)	QUR (mg)	NTZ (mg)	Particle Size (nm)	PDI	ZP (mV)	EE of QUR (%)	EE of NTZ (%)
**NT-1**	95	5	4	3	256.00 ± 0.70	0.288 ± 0.03	−18.6 ± 3.3	81 ± 0.11	90 ± 0.03
**NT-2**	87.5	12.5	4	3	205.12 ± 5.02	0.120 ± 0.01	−11.58 ± 0.5	76 ± 0.01	82 ± 0.01
**NT-3**	95	12.5	5	3	210.90 ± 3.67	0.155 ± 0.01	−15.06 ± 1.5	85 ± 0.02	88 ± 0.01
**NT-4**	80	12.5	5	3	186.60 ± 1.12	0.095 ± 0.02	−8.25 ± 0.1	72 ± 0.04	79 ± 0.10
**NT-5**	80	12.5	3	3	177.01 ± 1.36	0.092 ± 0.02	−8.95 ± 0.1	68 ± 0.04	81 ± 0.10
**NT-6**	95	12.5	3	3	209.95 ± 1.19	0.144 ± 0.02	−12.10 ± 1.4	77 ± 0.15	89 ± 0.01
**NT-7**	87.5	20	5	3	195.60 ± 0.19	0.102 ± 0.01	−9.78 ± 0.1	79 ± 0.03	78 ± 0.06
**NT-8**	87.5	20	3	3	190.80 ± 5.83	0.104 ± 0.01	−9.06 ± 0.2	66 ± 0.01	80 ± 0.02
**NT-9**	80	5	4	3	222.60 ± 4.10	0.246 ± 0.05	−16.10 ± 2.1	75 ± 0.01	83 ± 0.19
**NT-10**	87.5	5	5	3	241.25 ± 0.91	0.236 ± 0.01	−20.09 ± 0.3	67 ± 0.11	87 ± 0.01
**NT-11**	80	20	4	3	156.25 ± 5.83	0.085 ± 0.06	−7.96 ± 1.2	70 ± 0.01	76 ± 0.01
**NT-12**	95	20	4	3	202.20 ± 0.70	0.154 ± 0.01	−8.01 ± 0.6	80 ± 0.18	85 ± 0.03
**NT-13**	87.5	5	3	3	229.60 ± 0.55	0.216 ± 0.01	−17.23 ± 2.8	75 ± 0.02	88 ± 0.01

QUR: Quercetin, NTZ: Nitazoxanide, PDI: Poly dispersity index, ZP: Zeta potential, EE: Entrapment Efficiency, mV: Millivolt, nm: Nanometer. Data is represented as Mean ± Standard deviation (n = 3**)**.

#### 2.3.2 Preparation of NTZ-QUR-NTG

NTZ-QUR-NTG was prepared by incorporating NTZ-QUR-NT in medium molecular weight chitosan in order to make NTZ-QUR-NT appropriate for topical delivery. Briefly, 2 % chitosan powder was added into 2 mL of 1 % acetic acid solution under continuous stirring to prepare blank gel. Then, 3 mL of NTZ-QUR-NT were added into already prepared blank gel with consistent stirring to make the final NTZ-QUR-NTG [30].

### 2.4 Characterization of NTZ-QUR-NT

#### 2.4.1 Particle size, PDI, ZP analysis of NTZ-QUR-NT

NTZ-QUR-NT particle properties were examined under dynamic light scattering at 25 °C using a Zetasizer ZS 90 equipped with He–Ne laser which operate at 635 nm wavelength. The measurements were done with Zetasizer software of version 6.34 (Malvern Instruments, Worcestershire, UK) using a static scattering angle of 90° for cumulative analysis. NTZ-QUR-NT (10 μL) were dissolved in 1 mL of de-ionized water for analysis [31,32].

#### 2.4.2 Entrapment efficiency

The prepared NTZ-QUR-NT were centrifuged at 18746×*g* for 3 h using centrifuge apparatus (Hermle labortechnik, Z-216MK, Wehingen Germany) followed by the separation of the pellets from the free drug. A UV–visible spectrophotometer (Halo DB-20, Dynamica, UK) was used to observe the absorbance's at 335 and 256 nm, *λ*_max_ of NTZ and QUR respectively to determine the concentrations of the unentrapped drugs [33]. Equation given below was used to determine percentage entrapment efficiency (%EE) [34].

$$\%EE=\frac{\text{Total}\;\text{amount}\;\text{of}\;\text{drug}–\text{unentrapped}\;\text{drug}}{\text{Total}\;\text{amount}\;\text{of}\;\text{drug}\;\text{added}}*100$$

#### 2.4.3 Transmission electron microscopy (TEM)

The NTZ-QUR-NT were subjected to a morphological analysis by using TEM (Hitachi H- 7600; Tokyo, Japan) at a 100 kV accelerating voltage. To fix the particles on the carbon substrate, a tiny drop of sample was applied to the copper grid that was coated with carbon. The sample was negatively stained with 2 % phosphotungstic acid to adhere it to the carbon coating, followed by TEM examination [22,35].

#### 2.4.4 Fourier-transform infrared spectroscopy (FTIR)

FTIR (Nicolet-6700, Thermo Scientific, Lenexa, KS USA) was utilized to check the functional groups to identify any chemical interaction between QUR, NTZ, PL90G in NTZ-QUR-NT. All the samples were scanned at wavelength range from 400 to 4000 cm^−1^ and observations were analyzed by OMNIC™ software (version 7.3) [36,37].

#### 2.4.5 Deformability index of NTZ-QUR-NT

The deformability index (DI) was measured using extrusion. For that purpose, a filter with a 100 nm pore size was used to process the samples. The Zetasizer was used to measure the size of the NTZ-QUR-NT formulation before and after extrusion [29].

$$DI=\frac{Size\;after\;extrusion}{Size\;before\;extrusion}$$

### 2.5 Characterization of NTZ-QUR-NTG

#### 2.5.1 Organoleptic evaluation and pH measurement

Appearance, color, and homogeneity of the NTZ-QUR-NTG was examined physically and pH of the NTZ-QUR-NTG was evaluated by using digital pH meter (PH 700 EUTECH instruments, Aachen Germany).

#### 2.5.2 Rheology

Cone and plate Brookfield rheometer with a spindle CPA 42-Z (Brookfield Engineering Laboratories Inc., Middleborough, MA USA) was used for rheological evaluation of the NTZ-QUR-NTG. Four mL of gel was added to the plate of rheometer, and runs were carried out at temperature of 25 ^°^C with shear rates ranging from 1 to 100 s^−1^. A rheogram was plotted between shear rate and viscosity of the NTZ-QUR-NTG [38].

#### 2.5.3 Drug content

To determine the drug content of the NTZ-QUR-NTG, 5 mg of gel was added into 5 mL of phosphate buffer saline (PBS) and was kept for 24 h. After 24 h, NTZ-QUR-NTG and PBS mixture was stirred for 30 min by using magnetic stirrer (Eisco Scientific, North America). Lastly, this solution was filtered and absorbance was observed with UV–visible spectrophotometer [39].

#### 2.5.4 Spreadability

In order to measure the spreadability of the NTZ-QUR-NTG, a circle of 2 cm diameter was marked on a glass slide, and 500 mg gel was placed on it. The gel was covered with a second glass slide, and a 500 g weight was applied to it for 5 min. Then, increase in the gel diameter was noted which was considered as final area after spreading of the gel [40]. Lastly, the % spreadability was calculated by using this given formula:

$$\%\text{Spreadability}=\frac{\text{Final}\;\text{area}\;\text{after}\;\text{spreading}}{2\text{cm}}\times 100$$

### 2.6 Stability study

NTZ-QUR-NT and NTZ-QUR-NTG stability study was conducted at different temperatures i.e. 4 °C and 25 °C for a period of six months to evaluate the effects of storage conditions. At certain time points (0, 1, 3 and 6 months), one sample each of NTZ-QUR-NT and NTZ-QUR-NTG was analyzed in terms of particle size PDI, ZP, color change, homogeneity and phase separation [41,42].

### 2.7 In-vitro drug release and kinetic study

The drug release profiles of the NTZ-QUR-NT and NTZ-QUR-NTG were evaluated using dialysis membrane method and were compared with pure NTZ and QUR dispersion. To imitate the pH of blood and macrophages, a drug release study was performed at pH 7.4 and 5.5, respectively. All the formulations were put into dialysis bags and suspended in beakers containing 100 mL of respective release media (PBS of pH 7.4 and 5.5) in a shaker water bath which was maintained at 37 °C. To maintain sink conditions, a specific volume of samples was removed at regular intervals (0.25, 0.5, 1, 2, 4, 6, 8, 12 and 24 h) and replaced with a comparable volume of fresh buffer. Evaluation of withdrawn samples were done spectrophotometrically, and graphs were plotted between % cumulative release of drug and time. Moreover, DD-solver was used to apply different kinetic models to determine the most fitting model which explained the release kinetics of the drugs from NT and NTG [24].

### 2.8 Ex-vivo skin permeation and deposition study

Horizontal Franz diffusion cell apparatus was utilized to perform ex-vivo permeation experiment. The Franz diffusion cell having 0.78 cm^2^ permeation area and a 5.2 mL receiving compartment was utilized. The system's temperature was kept at 32 °C and recently excised rat skin was put between the donor and receiving compartments. Test formulations were inserted in the donor compartment, which have non-occlusive hydration system. Samples from the receiving compartment were taken at intervals of 0.5, 1, 2, 3, 4, 6, 8, 12 and 24 h while replacing the sample with an equivalent volume of PBS [43]. Finally, the graph was plotted showing the cumulative drug permeation per unit area vs time.

After ex-vivo permeation investigation, the rat skin was dismounted and to get rid of any residual formulation skin sections were rinsed with distilled water and blot dried [44]. The SC was separated from the remaining skin layers utilizing the tape stripping technique. With 15 to 20 layers of adhesive tape, the skin parts were stretched and peeled. Drugs were released from tape strips by crushing the strips in methanol at 37 °C and the spectrophotometer was used to detect them. Following tape removal, the remaining skin part was chopped into small pieces, crushed, and homogenized in methanol. The drugs were then quantified using a UV–visible spectrophotometer [45].

### 2.9 Skin structure evaluation after treatment

FTIR was used to assess the changes in epidermal lipid organization after applying NTZ-QUR-NTG and its comparison was done against normal skin. In this study, the epidermis was inserted between the donor and receptor compartments following complete separation of skin layer. NTZ-QUR-NTG was applied to skin layers for 4 h to investigate drugs penetration. In order to analyze the functional group of skin membrane at wave number 4000 - 400 cm^−1^, the epidermal layer was removed after 4 h, soaked in PBS to remove the remains of formulation, dried, and then observed by FTIR [29].

### 2.10 Skin irritation study and histopathological study

The Draize scoring system was used for skin evaluation after treatments. The rats were categorized into three groups (positive, negative and treated group). Untreated rats were kept in negative control group and 0.8 % formalin treated rats were considered as positive control group and in treated group 0.5 g of NTZ-QUR-NTG was applied. Rats skin was observed for any sign of redness or edema and scoring was done at different time points including 1 h, 24 h, 48 h and 72 h. The results were further verified by histopathological examination at completion of the study (72 h). Cryostat microtome slices of skin samples were prepared, followed by microscopic inspection [46,47].

### 2.11 Qualitative macrophage uptake study

Peritoneal macrophages (PMs) were used for uptake study. To extract the PMs, rat's peritoneal cavity was cautiously injected with 2 mL of sterile thioglycolate (3 % w/v) to avoid the bladder. After allowing the inflammatory response to last for four days, the rats were euthanized. The peritoneal cavity was then filled with 4 mL of ice cold RPMI media and then peritoneal exudate were collected. The exudates were centrifuged for 10 min at 4277×*g* and the pellet was suspended in RPMI medium that had been supplemented with 10 % fetal bovine serum (FBS) and 100 μg/mL streptomycin sulphate. In culture well plates, PMs had been incubated with rhodamine-labeled NT (washed three times prior to the experiment) at a cell density of 2 × 10^4^ cells. For comparisons, plain rhodamine solution was used as control. After 30 min in a CO_2_ incubator, all samples were withdrawn to remove non-adherent cells with washing in PBS and adhered cells were examined under a fluorescence microscope [48].

### 2.12 Quantitative macrophage uptake study

The NTZ-QUR-NT and NTZ-QUR dispersion were planted in culture well plates in order to quantitatively measure the cellular absorption of NT (Supplemental Fig. S2). After 24 h, the plate was removed from incubator and PBS was cleaned from them. Adherent cells were scraped from slides, collected and centrifuged. After that, in methanol the pellets were re-dispersed and sonicated for 5 min followed by centrifugation. Lastly, the concentration of NTZ and QUR was determined using an UV–visible spectrophotometer [45].

### 2.13 Cell viability and toxicity assay

To determine the cytotoxicity of NTZ-QUR dispersion and NTZ-QUR-NT, previously isolated macrophages were put through a (4, 5-dimethylthiazol-2-yl)-2, 5-diphenyl tetrazolium bromide (MTT) experiment. The MTT assay uses mitochondrial succinate dehydrogenase to turn a yellow-colored tetrazolium component into blue, insoluble formazan crystals. Isolated macrophages were seeded into a 96-well plate for the MTT assay, which was then incubated for 24 h at 37 °C in a 5 % CO_2_ incubator. NTZ-QUR dispersion and NTZ-QUR-NT were introduced to the well in varying concentrations (as above) and incubated for 24 h. After 24 h, wells were filled with 20 μL of MTT solution and incubated for 4 h to create formazan crystals, which were subsequently liquefied with 100 μL of DMSO**.** Then, absorbance was determined at a wavelength of 540 nm using a microplate reader (BioTek, USA) [49,50]. Percent cell viability was calculated by using following equation:

$$\%\text{Cell}\;\text{viability}=\frac{\text{Absorbance}\;\text{of}\;\text{sample}}{\text{Absorbance}\;\text{of}\;\text{control}}\times 100$$

### 2.14 In-vitro anti-leishmanial assay against *L. Tropica*

An in-vitro anti-leishmanial investigation was carried out using MTT assay. At 24 °C, *L*. tropica promatigotes were grown in RPMI medium that contained 10 % fetal bovine serum (FBS), 100 IU/mL penicillin, and 100 μg/mL streptomycin sulphates. In our study we used late stationary phase promastigotes from 3–5-day growth culture (early passage). Neubauer hemocytometer was used to count the promastigotes that were seeded at a density of 1 × 10^6^ promastigotes/mL in each well of 96-well plate containing 20 μL samples. DMSO and amphotericin B were regarded as negative and positive controls, respectively. The culture plates underwent a 72 h incubation period at 24 °C. Each culture plate received a 20 μL pre-filtered MTT solution with a concentration of 4 mg/mL formulated in distilled water. The plates were again incubated for 24 h at 24 °C. After 24 h, supernatant from plates was carefully removed without disturbing the sediment that contained colorful formazan. Formazan crystal dissolution was accomplished by adding DMSO (100 μL) to the sediment. After 1 h, the absorbance at a wavelength of 540 nm was measured using microplate reader. The IC_50_ were calculated by using GraphPad Prism® software (version 5) [30].

### 2.15 Determination of combination index

Combination index (CI) was calculated to assess the cumulative therapeutic impact resulting from combination of NTZ and QUR. The value of CI < 1 shows synergistic behavior, CI > 1 shows antagonistic behavior, while CI value equal to 1 shows additive effect [51]. CI was calculated by using following formula:

$$CI=\frac{{IC}_{50}\left(A+B\right)}{{IC}_{50}\left(A\right)}+\frac{{IC}_{50}\left(A+B\right)}{{IC}_{50}\left(B\right)}$$

where the IC_50_ values for drugs separately are denoted by IC_50_ (A) and IC_50_ (B). While IC_50_ (A + B) represents the IC_50_ of drugs combination.

### 2.16 In-vivo anti-leishmanial effect

In-vivo anti-leishmanial effect of the NTZ-QUR-NTG was investigated and compared with NTZ-QUR-G (conventional gel) and untreated control groups, using *BALB/c* mice. The mice were injected with 10 μL saline suspension of *L. tropica* promastigotes subcutaneously into dermis of the right ear. Mice were randomly divided into three groups (n = 6), one of which was not treated with any formulation. The other groups received NTZ-QUR-NTG and NTZ-QUR-G topically after development of the lesion (in 2 weeks of the study). The lesion size was measured using a digital caliper at week 3, 4, 5, 6 and 8 to find alteration in the infected and uninfected contralateral ears [46]. The data was plotted to understand the antileishmanial effect by reduction in the lesion size.

### 2.17 Statistical analysis

Statistical analysis and optimization for formulation development were carried out using Design Expert® Software (version 12). Moreover, GraphPad Prism® (version 5) and Microsoft Excel 365 (version 2010) were used to examine all other results. One-way ANOVA and Student t-test were also used for statistical analysis. Statistical data was considered significant for analysis with a *p*-value less than 0.05. Additionally, the results were all reported as Mean ± Standard deviation.

## 3 Results

### 3.1 Statistical optimization of NTZ-QUR-NT employing Box-Behnken design

**Fig. 3:**
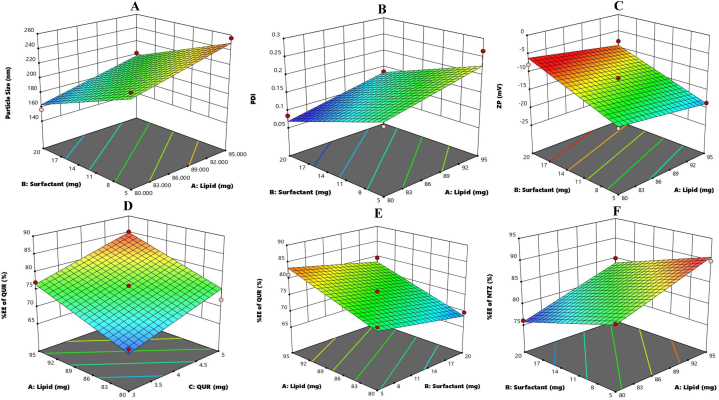
Optimization analysis of different variables on NTZ-QUR-NT via Box-Behnken design: **(A)** Particle size; **(B)** Polydispersity index (PDI); **(C)** Zeta potential (ZP); **(D & E)** % Entrapment efficiency (EE) of QUR and **(F)** %EE of NTZ.

Optimization of the NTZ-QUR-NT was done by using Box-Behnken design of the Design Expert® software (Stat-Ease Inc., USA). Lipid (PL90G), surfactant (Tween® 80) and drug (QUR) were selected as independent variables while particle size, PDI, ZP and %EE were kept as dependent variables. The Box-Behnken design has generated 13 experimental runs and the assessed response variables are presented in Table 1. Influence of lipid, surfactant and QUR concentration on particle size, PDI, ZP and EE was determined using this design. The results of Box-Behnken design analysis in form of R^2^ value, adjusted R^2^ value, predicted R^2^ value and adequate precision of each response variable is stated in Supplemental Table S1. The product parameter interactions were clearly explained by 3D response surface graphs as presented in Fig. 3.

#### 3.1.1 Effect of independent factors on particle size

All the 13 formulations suggested by Design Expert® software were prepared and evaluated, having particle size fluctuating between 156.25 ± 5.83 nm and 256.50 ± 0.70 nm (shown in Table 1). Lipid and surfactant concentrations have significant (*p* = 0.0002, 0.0001) effect on NTZ-QUR-NT particle size. On the other hand, QUR concentration has non-significant (*p* = 0.2860) effect on particle size of the NTZ-QUR-NT. Keeping surfactant and QUR concentration constant, when the lipid content was varied from 80 mg to 95 mg the particle size of NTZ-QUR-NT was increased from 222.60 nm to 256.00 nm as seen in formulation no. NT-9 and NT-1, respectively. However, the trend of surfactant concentration was contrary to the lipid concentration. Particle size of NTZ-QUR-NT were reduced with surge in surfactant concentration as illustrated in Fig. 3A. As evident from NT-1 and NT-12, the NTZ-QUR-NT particle size decreased from 256.00 nm to 202.20 nm when surfactant content was varied from 5 mg to 20 mg.

#### 3.1.2 Effect of independent factors on PDI

Linear model was found to be significant in case of PDI after applying ANOVA test. Herein, the lipid and surfactant were found controlling factors, having *p* values 0.0008 and 0.0001, correspondingly. As evident from the results in Fig. 3B and Table 1, when surfactant quantity was elevated from 5 mg to 20 mg, PDI was decreased from 0.25 (NT-9) to 0.09 (NT-11). On the contrary, increase in lipid content from 80 mg to 95 mg cause surge in PDI from 0.10 (NT-4) to 0.16 (NT-3). However, the concentration of QUR has not markedly affected the PDI of the NTZ-QUR-NT.

#### 3.1.3 Effect of independent factors on ZP of NTZ-QUR-NT

Both lipid and surfactant have significant effect (*p* value 0.0345, 0.001) on ZP of the formulation as shown in Fig. 3C. The negative charge on formulation is attributed to the lipid (PL90G) which forms double layer of nano vesicles [46]. As noticeable from Table 1, when concentration of the lipid was increased from 80 mg to 95 mg, there was escalation in the negative charge on particles from −8.1 mV (NT-4) to −15.1 mV (NT-3). In contrast, when the concentration of surfactant (Tween® 80) was increased from 5 mg to 20 mg, there was significant decrease in negative charge on the particles from −20.1 mV (NT-10) to −9.8 mV (NT-7). The reduction in negative value of ZP was observed with increase in Tween® 80 concentration.

#### 3.1.4 Effect of independent factors on %EE of NTZ and QUR

All the independent factors: lipid, surfactant and QUR have significant effect on the entrapment of NTZ and QUR as shown in Figs. 3D–2F. As the concentration of lipid increased from 80 mg to 95 mg, %EE of NTZ was enhanced from 76 % (NT-11) to 85 % (NT-12) and for QUR from 69.64 % to 79.53 %. On the contrary, when content of surfactant increased from 5 mg to 20 mg %EE of NTZ was decreased from 83 % (NT-9) to 76 % (NT-11) and QUR from 75 % to 70 % as shown in Table 1. Lastly, 4 % increase in %EE of QUR was observed with surge in the concentration of QUR from 3 mg to 5 mg.

#### 3.1.5 Prediction of the optimized formulation

**Fig. 4:**
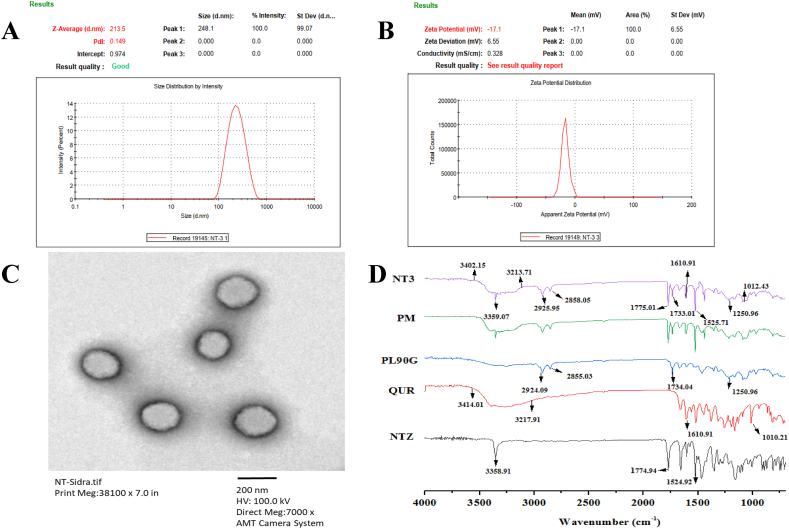
Evaluation of NTZ-QUR-NT **(A)** Particle size & PDI; **(B)** Zeta potential; **(C)** Transmission electron micrograph (TEM) micrograph (7000×) **(D)** FTIR analysis of NTZ, QUR, PL90G, PM and NTZ-QUR-NT observed in the range of 4000–400 cm^−1^.

To find an optimized formulation of NT, multiple response optimizations were utilized that fulfill our requirement to achieve minimum vesicle size, PDI, optimum ZP and maximum %EE. The optimized formulation was NT-3 as predicted by Box-Behnken design with particle size 210.90 ± 3.67, PDI 0.16 ± 0.009 and ZP -15.1 ± 1.48 as shown in Fig. 4A and **B**. With an acceptable particle size NT-3 also showed good particles segregation and stability with high entrapment of NTZ and QUR.

### 3.2 TEM analysis of NTZ-QUR-NT

TEM analysis of NTZ-QUR-NT was performed. The TEM image showed that NT had uni-lamellar and spherical shape. The particles’ size was less than 250 nm with uniform distribution as shown in Fig. 4C.

### 3.3 Deformability index (DI) of NTZ-QUR-NT

DI value of the NTZ-QUR-NT was used to evaluate its stress-dependent flexibility and squeezing capacity [52]. NTZ-QUR-NT deformability index was found to be 0.9264 ± 0.042, indicating deformable nature of NT.

### 3.4 FTIR analysis to assess the compatibility of excipients with drugs

FTIR analysis of NTZ, QUR, PL90G, physical mixture (PM) and NTZ-QUR-NT was performed to check the functional groups and chemical interaction among the constituents of the formulation. FTIR spectrum of NTZ, QUR and PL90G showed characteristic peaks as presented in Fig. 4D. Stretching at 3358.91 cm^−1^,1774.94 cm^−1^ and 1524.92 cm^−1^ correspondingly represents N–H, C]O and N]O functional groups of NTZ [53]. Vibrations at 3402.15 cm^−1^ and 3213.71 cm^−1^ showed the presence of hydroxyl groups (OH) of QUR. Peaks at 1610.91 cm^−1^ and 1010.21 cm^−1^ represents C]C bond stretching of alkyne group and C–C bending of alkyl group of QUR [54]. Characteristic band at 2924.09 cm^−1^ and 2855.03 cm^−1^ exhibits hydrocarbons stretching of PL90G. Our results demonstrated all the specific peaks of NTZ, QUR and PL90G which clearly showed the compatibility of drugs and excipients in formulation [55,56].

### 3.5 NTZ-QUR-NTG physicochemical characterization

Physicochemical characterization of NTZ-QUR-NTG was carried out and it was observed that NTZ-QUR-NTG appearance was opaque yellowish in color with uniform homogeneity. The pH of NTZ-QUR-NTG was 5.8 ± 1.56 which was within 4–6, the usual range for the pH of skin [57,58]. The spreadability of NTZ-QUR-NTG was 310.50 ± 3.5 %, compatible for topical use as high spreadability and faster dissemination are characteristics of an excellent gel [59]. The drug content of NTZ and QUR was found to be 98.45 ± 1.17 % and 97.86 ± 1.39 %. All observed parameters of NTZ-QUR-NTG are presented in Supplemental Table S2. Additionally, the impact of shear rate upon viscosity was examined using a Brookfield viscometer and the results showed that viscosity of gel decreases with increase in shear rate. Supplemental Fig. S3 depicts the shear thinning and pseudoplastic behavior of the gel.

### 3.6 Stability studies

The stability study of NTZ-QUR-NT and NTZ-QUR-NTG is shown in Table 2, Table 3. NTZ-QUR-NT were assessed for particle size, PDI and zeta potential. Stability data showed that there was non-significant increase in NTZ-QUR-NT particle size from 213.50 ± 3.67 nm to 219.26 ± 4.14 nm (at 4 °C) and 222.62 ± 5.61 (at 25 °C), respectively. Similarly, there was negligible variation in PDI and ZP during the storage period. The initial PDI and ZP values of optimized formulation were 0.16 ± 0.009 and −17.1 ± 1.48 mV. After six month, it was found to be 0.22 ± 0.052 and −15.4 ± 0.07 correspondingly. NTZ-QUR-NTG was also analyzed in terms of precipitation, drug content and physical appearance. The stability study findings showed no meaningful change in drug content of NTZ and QUR in NTZ-QUR-NTG. No change in color or precipitation was observed in NTZ-QUR-NTG during period of six month.

**Table 2:** Stability data of NTZ-QUR-NT.

Time period	At temperature 4 ± 2 °C			At temperature 25 ± 2 °C		
	Particle size (nm)	PDI	ZP (mV)	Particle size (nm)	PDI	ZP (mV)
**0 day**	213.50 ± 3.67	0.155 ± 0.01	−17.10 ± 1.4	213.50 ± 3.67	0.155 ± 0.01	−17.10 ± 1.4
**1 month**	215.71 ± 2.25	0.164 ± 0.01	−16.89 ± 1.5	217.21 ± 4.33	0.172 ± 0.04	−15.81 ± 1.2
**3 month**	217.04 ± 2.64	0.167 ± 0.01	−16.07 ± 1.5	219.17 ± 5.09	0.184 ± 0.09	−15.92 ± 1.6
**6 month**	217.26 ± 4.14	0.202 ± 0.01	−15.80 ± 1.4	222.62 ± 5.61	0.218 ± 0.05	−15.39 ± 0.1

Data is represented as Mean ± Standard deviation (n = 3). PDI: Polydispersity index, ZP: Zeta Potential, mV.

Millivolt, nm: Nanometer.

**Table 3:** Stability data of NTZ-QUR-NTG.

Time period	At temperature 4 ± 2 °C				At temperature 25 ± 2 °C			
	%DC of NTZ	%DC of QUR	Physical appearance	Precipitation	%DC of NTZ	%DC of QUR	Physical appearance	Precipitation
**0 day**	98.45 ± 1.17	97.86 ± 1.39	Uniform	No	98.45 ± 1.17	97.86 ± 1.39	Uniform	No
**1 month**	98.32 ± 1.07	97.72 ± 1.16	Uniform	No	98.29 ± 1.09	97.65 ± 1.12	Uniform	No
**3 month**	98.11 ± 1.98	97.51 ± 1.38	Uniform	No	98.22 ± 1.18	97.44 ± 1.88	Uniform	No
**6 month**	98.10 ± 1.09	97.40 ± 1.26	Uniform	No	97.84 ± 1.39	97.10 ± 1.21	Uniform	No

Data is represented as Mean ± Standard deviation (n = 3). DC: Drug content.

### 3.7 In-vitro release study of NTZ and QUR at pH 5.5

**Fig. 5:**
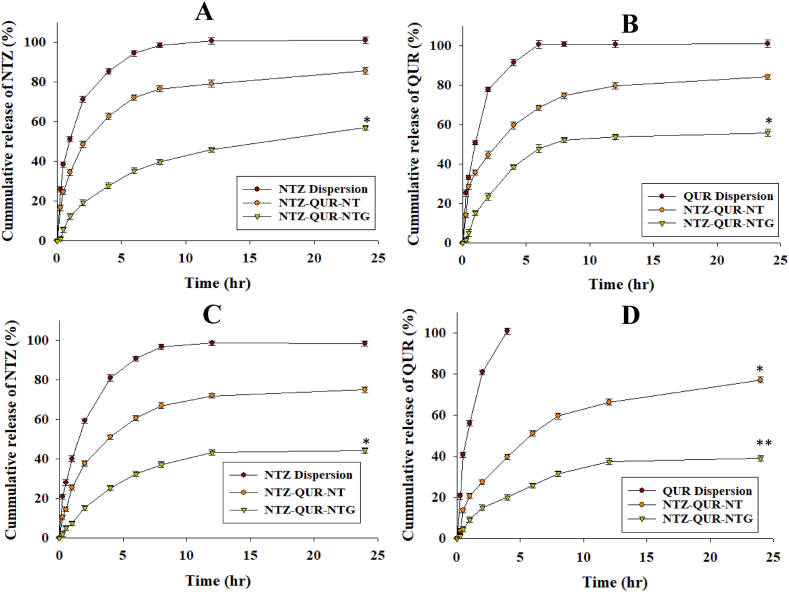
In vitro release pattern at pH 5.5 **(A)** NTZ; **(B)** QUR; and at pH 7.4 **(C)** NTZ; **(D)** QUR. Data is represented as Mean ± Standard deviation (n = 3), **p < 0.01 as compared to NTZ and QUR dispersion and NTZ-QUR-NT, *p < 0.05 as compared to NTZ and QUR dispersion.

The release study was conducted at pH 5.5, mimicking skin macrophage endosomes and in-vitro release pattern of both the drugs (NTZ & QUR) as shown in Fig. 5 (A) and **5 (B).** NTZ dispersion displayed 85 % NTZ release in 4 h, reaching 100 % by 12 h, whereas, NTZ-QUR-NT released 62 % NTZ in 4 h, reaching 80 % in 24 h. In contrast, NTZ-QUR-NTG showed slower release of the NTZ as 27 % and 56 % was released in 4 h and 24 h, respectively. QUR dispersion achieved 100 % QUR release in 6 h. NTZ-QUR-NT released 67 % QUR in 6 h followed by 85 % release in 24 h. In contrast, NTZ-QUR-NTG released 38 % and 60 % QUR released in 6 h and 24 h, respectively.

### 3.8 In-vitro release study of NTZ and QUR at pH 7.4

In vitro release study of QUR and NTZ was also performed at physiological pH as presented in Fig. 5 (C) and **5 (D)**. Around 80 % of NTZ was released from NTZ dispersion in the first 4 h. Unlike which, 52 % and 30 % of NTZ was released from NTZ-QUR-NT and NTZ-QUR-NTG, respectively within 4 h followed by 78 % and 49 % release after 24 h. While 100 % of QUR was released from QUR dispersion, but only 40 % and 25 % was released NTZ-QUR-NT and NTZ-QUR-NTG in 3 h, respectively followed by 76 % and 42 % QUR release in 24 h.

### 3.9 Release kinetic study

To analyze the release pattern of QUR and NTZ from NT and NTG, different kinetic models (First order, zero order, Korsmeyer-Peppas, Hixon-Crowell, first order, Higuchi) were applied by utilizing DD-solver. In Table 4, R^2^ value of all models are stated. By analyzing R^2^ values of all models Korsmeyer-Peppas was considered as most fitting for release of both drugs from NT and Higuchi for NTG. Their kinetic model graphs are presented in Supplemental Fig. S4. As the values of n were less than 0.45 in case NTZ-QUR-NT and greater than 0.45 in case of NTZ-QUR-NTG which suggested that the release of QUR and NTZ was through fickian diffusion from NT and non-fickian from NTG.

**Table 4:** R^2^ values of kinetic models.

Release Model	R^2^ Values of Release Model			
	Nitazoxanide		Quercetin	
	NTZ-QUR-NT	NTZ-QUR-NTG	NTZ-QUR-NT	NTZ-QUR-NTG
**Zero order**	−0.8236	0.5248	−0.4158	0.4807
**First order**	0.7890	0.8160	0.8519	0.8374
**Higuchi**	0.6412	0.9724	0.7728	0.9511
**Hixon-Crowell**	0.6722	0.7398	0.7538	0.7516
**Korsmeyer-Peppas(n)**	0.9154 (0.292)	0.9596 (0.472)	0.9387 (0.323)	0.9369 (0.464)

### 3.10 Ex-vivo skin permeation and deposition studies

**Fig. 6:**
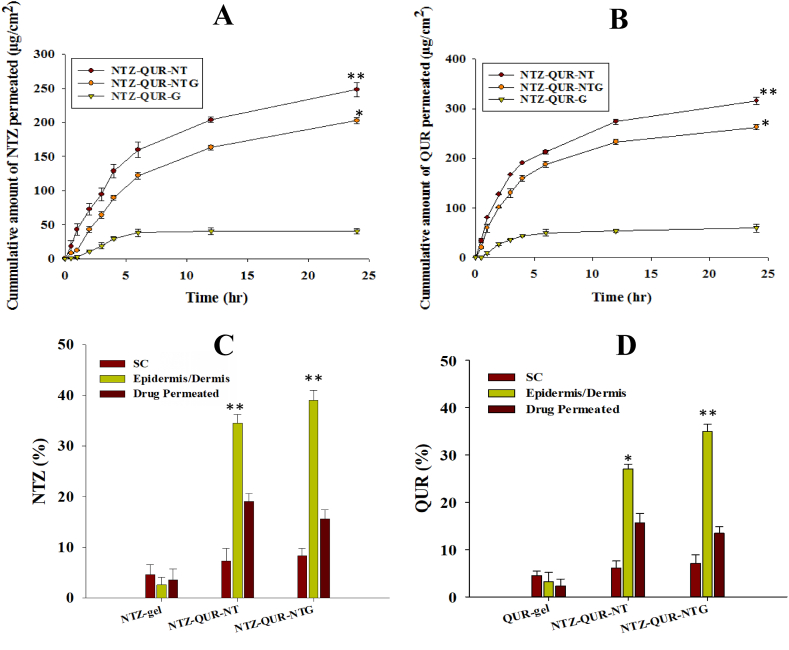
Ex-vivo permeation of **(A)** NTZ; **(B)** QUR and Skin deposition of **(C)** NTZ and **(D)** QUR. Data are represented as Mean ± Standard deviation (n = 3), **p < 0.01 compared to NTZ and QUR dispersion and NTZ-QUR-NT, *p < 0.05 as compared to NTZ and QUR gel.

Ex-vivo permeation analysis of all tested formulations are illustrated in Fig. 6 (A) and 6 **(B)**. The cumulative amount of NTZ and QUR permeated from per unit area of rat skin from NTZ-QUR- NT was 248.23 μg/cm^2^ and 316.26 μg/cm^2^, respectively. While permeation of both drugs from NTZ-QUR-NTG was 202.85 μg/cm^2^ and 262.72 μg/cm^2^ which was little less than NT due to controlling nature of the chitosan gel. However, the permeation of NTZ and QUR from plain gels was only 40.54 μg/cm^2^ and 59.37 μg/cm^2^. By comparing the results and finding the enhancement ratios, it was observed that the permeation of NTZ and QUR from our optimized formulation were increased 5-folds and 4-folds as compared to the plain gel. Additional permeation parameters are presented in Table 5. Moreover, deposition study showed that 38.74 % and 34.97 % of NTZ and QUR respectively were retained in deeper layers of skin (epidermis and dermis) from transferosomal gel whereas only 3.21 % and 2.75 % of both drugs were retained from plain NTZ and QUR gel. Deposition graphs of NTZ and QUR presented in Fig. 6 (C) and **6 (D)**.

**Table 5:** Skin permeation parameters of NTZ and QUR.

Formulation	Nitazoxanide		
	Total amount of drug permeated in 24 h (μg/cm^2^)	Flux J_max (_μg/cm^2^/h)	Enhancement ratio
**NTZ-gel**	40.541	1.689	1
**NTZ-QUR-NT**	248.23	10.34	6.12
**NTZ-QUR-NTG**	202.85	8.45	5.02
	**Quercetin**		
**QUR-gel**	59.37	2.47	1
**NTZ-QUR-NT**	316.26	13.19	5.34
**NTZ-QUR-NTG**	262.72	10.94	4.42

### 3.11 Skin structure evaluation after treatment

**Fig. 7:**
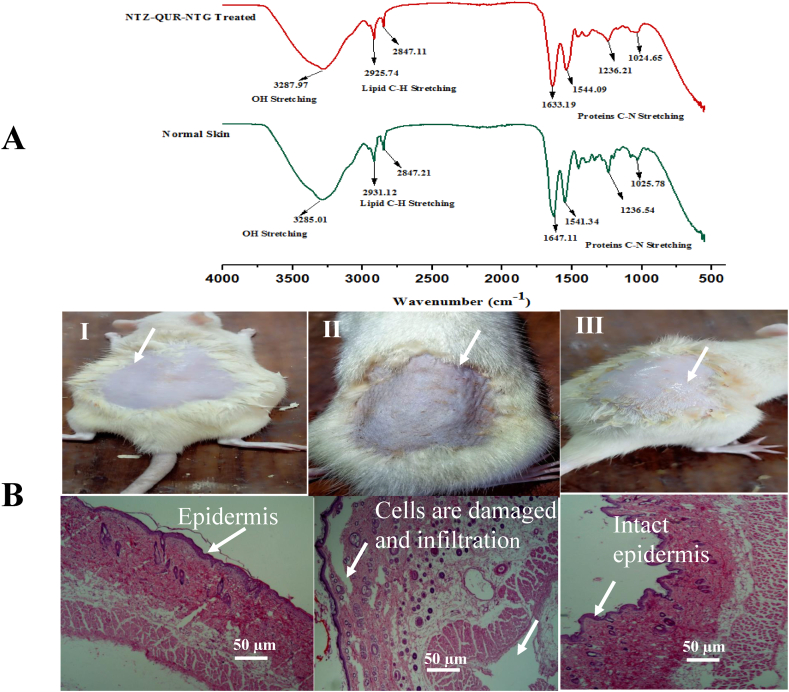
**(A)** shows comparison of FTIR of treated and untreated skin (epidermal layer of rat skin treated with NTZ-QUR-NTG extracted, immerse in PBS to remove formulation, dried and subjected to FTIR (4000-650 cm^−1^) **(B)** Histopathological analysis of **(I)** Normal skin with no sign of inflammation **(II)** Formalin (0.8 %) treated skin which shows inflammation, redness and under microscope shows infiltration and **(III)** NTZ-QUR-NTG treated skin shows intact dermis with no inflammation.

After treatment with NTZ-QUR-NTG, FTIR analysis of the skin was performed to evaluate any structural alterations and compared it with normal skin (Fig. 7A). The characteristic peak of C–H bond stretching was observed in both normal and NTZ-QUR-NTG treated skin at 2925.74 cm^−1^and 2931.12 cm^−1^, respectively. FTIR analysis showed that the integrity of the skin hydrocarbon lipid bilayers was preserved after application of NTZ-QUR-NTG. For normal skin, amide group peaks were seen at 1633.19 cm^−1^ and 1544.09 cm^−1^, while for skin treated with NTZ-QUR-NTG, peaks were seen at 1647.11 cm^−1^ and 1541.34 cm^−1^, indicating no significant change in skin structure.

### 3.12 Skin irritation and histopathology study

A skin irritation investigation using NTZ-QUR-NTG was conducted to verify safety and the results were compared with the formalin [60]. The Draize scoring method was used for evaluation of skin irritation test and results showed that NTZ-QUR-NTG was non-irritant as there was no difference in scoring of normal skin and NTZ-QUR-NTG treated skin. The Draize scoring is presented in Supplemental Table S3. The histopathology investigation showed no evidence of infiltration or loosened collagen fibers in normal rats skin (Fig. 7BI), however, inflammation and infiltration of the skin treated with 0.8 % formalin was observed (Fig. 7BII). Nevertheless, the epidermal layer was found to be unaltered, intact with no sign of inflammation as shown in Fig.7BIII. The outcomes were identical to what was seen with normal skin.

### 3.13 Qualitative macrophage uptake study

**Fig. 8:**
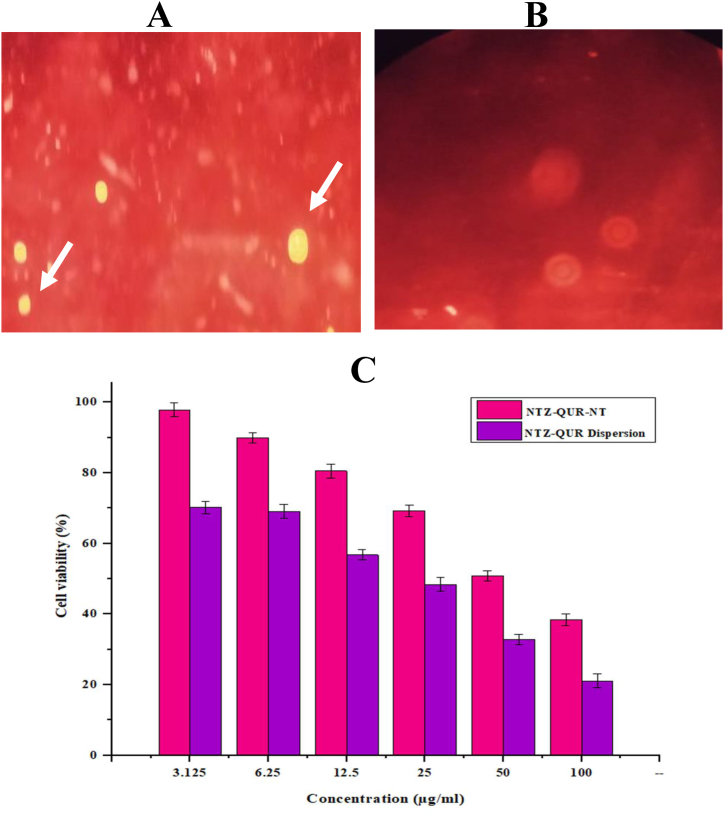
Macrophage uptake study: **(A)** Rhodamine loaded NT treated macrophages; **(B)** Rhodamine solution treated macrophages and **(C)** Cell viability assay of the NTZ-QUR-NT and NTZ-QUR dispersion (n = 3).

A macrophage uptake test was conducted to analyze the macrophage targeting potential of NT. The results showed intense internalized fluorescence after 1 h of incubation with rhodamine loaded NT, as presented in Fig. 8A, indicating its passive targeting on macrophages. On the other hand, no fluorescence was seen in Rhodamine solution which was used as a control (Fig. 8B).

### 3.14 Quantitative macrophage uptake study

The quantitative macrophage uptake investigation was used to confirm the qualitative evaluation of macrophage internalization. The findings revealed that more NTZ-QUR-NT were engulfed by macrophages. More specifically, 78.44 **±** 0.95 μg of NTZ and 61.14 **±** 1.23 μg of QUR from NTZ-QUR-NT were engulfed by 2 × 10^4^ macrophages. While in case of NTZ-QUR dispersion only 8.07 **±** 1.19 μg of NTZ and 5.41 **±** 1.54 μg of QUR were taken by 2 × 10^4^ macrophages. In comparison to NTZ-QUR dispersion, NTZ-QUR-NT cell internalization was almost 10-folds higher.

### 3.15 Cell viability and toxicity assay

Cell viability assay was also carried out on macrophages previously collected from rat abdominal cavity. It was found that, cell viability percentage of NTZ-QUR-NT was higher at each concentration as compared to NTZ-QUR dispersion. Such as, at concentration of 3.125 μg/mL, 98 % of cells were vial while in case of NTZ-QUR dispersion the viability of cells was 70 %. The significant difference of both groups can be seen in Fig. 8C. NTZ-QUR-NT and NTZ-QUR dispersion CC_50_ values were calculated by using GraphPad prism®. The results showed that NTZ-QUR-NT have 71.95 ± 3.32 % CC_50_ value and NTZ-QUR dispersion have 49.77 ± 2.15 % CC_50_, which indicated that NTZ-QUR-NT is safer to use as compared to NTZ-QUR dispersion [46].

### 3.16 In-vitro anti-leishmanial assay against *L. Tropica*

The in-vitro anti-leishmanial assay was performed to check the IC_50_ value of NTZ-QUR-NT and to compare it with NTZ-NT, QUR-NT, NTZ dispersion, QUR dispersion and NTZ-QUR dispersion (Table 6). IC_50_ values of plain NTZ and QUR dispersions alone were 29.67 ± 1.43 μg/mL and 43.72 ± 1.52 μg/mL, respectively. While the IC_50_ of NTZ and QUR in combination was 19.66 ± 1.17 μg/mL. The IC_50_ values of NTZ and QUR were reduced into 8.35 ± 0.95 μg/mL and 14.91 ± 1.09 μg/mL when separately loaded in NT. Moreover, the IC_50_ value was significantly reduced to 3.15 ± 0.89 μg/mL when the combination of both drugs was loaded into NT. IC_50_ date of formulations is shown in Table 6. The IC_50_ value of NTZ-QUR-NT against *L. tropica* promastigotes was 6 times lower than NTZ-QUR dispersion and 2.65 and 4.73 times as compared to NTZ-NT and QUR-NT, respectively. The CI value of NTZ-QUR-NT was found to be 0.58 which showed synergistic behavior of both drugs loaded in NT.

**Table 6:** In-vitro antileishmanial assay against *Leishmania tropica*.

Formulation	IC50 value (μg/mL)
NTZ-QUR-NT	3.15 ± 0.89
NTZ-NT	8.35 ± 0.95
QUR-NT	14.91 ± 1.09
NTZ-QUR dispersion	19.66 ± 1.17
NTZ dispersion	29.67 ± 1.43
QUR dispersion	43.72 ± 1.52

Data is represented as Mean ± Standard deviation (n = 3).

### 3.17 In-vivo anti-leishmanial effect

**Fig. 9:**
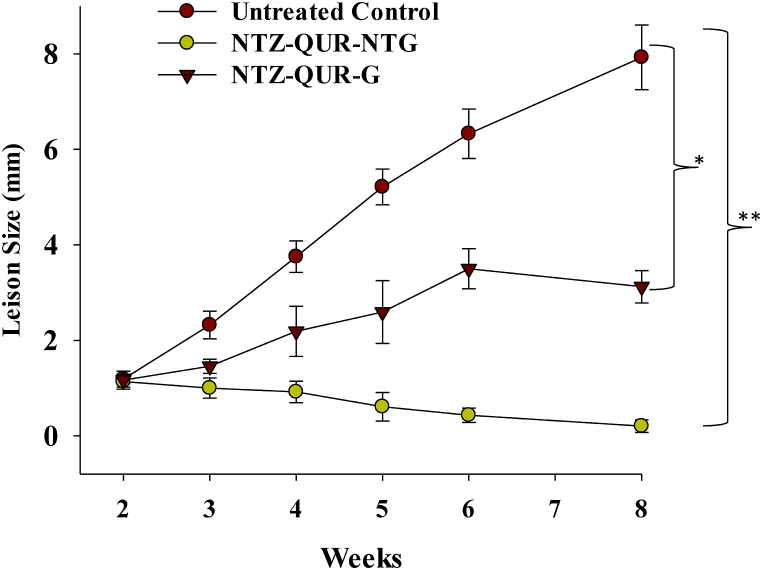
In-vivo antileishmanial analysis of NTZ-QUR-NTG and its comparison with NTZ-QUR-G and untreated mice groups. Data was taken in sextuplicate (n = 6). *p < 0.01 as compared to untreated group. *p* < 0.001 as compared to untreated and NTZ-QUR-G groups.

The results of in-vivo anti-leishmanial assay is reported as Fig. 9. A significantly enhanced lesion size (7.9 ± 0.6 mm) was observed for untreated control mice group after 8 weeks. However, the lesion size of the NTZ-QUR-G was found to be (3.1 ± 0.3 mm), which was meaningfully smaller as compared to untreated group. Nevertheless, the NTZ-QUR-NTG treated group revealed the lowest lesion size (0.2 ± 0.1 mm), demonstrating the efficacy of the formulation on all the testing groups, over a period of 8 weeks study.

## 4 Discussion

NTZ-QUR-NT were optimized using Box-Behnken design of Design Expert® software. Initially, through evaluation of the published literature minimum and maximum ranges of independent factors were fixed. PL90G, Tween® 80 and QUR were nominated as independent variables whereas, particle size, PDI, ZP and %EE were used as dependent variables. For dermal delivery in cutaneous *Leishmaniasis* the particles size is considered as a key factor. For optimal macrophage uptake in dermal skin layer the particle size should not be less than 100 nm and greater than 300 nm [45]. Particles less than 100 nm may escape from the engulfment by macrophages. However, particle size between 100 and 300 nm is ideal for macrophage targeting and penetration through stratum corneum (SC) [46]. With increase in lipid concentration the particle size of NTZ-QUR-NT was increased, similar behavior was reported in various other studies [30,61]. This increase in size could be due to increase in densely packed vesicular lipid bilayers [62]. Furthermore, as previously reported, the increase in lipid concentration may result in the development of multilamellar vesicles with larger diameters [63]. Nevertheless, the decrease in size because of higher Tween® 80 concentrations in NTZ-QUR-NT is due to its ability to lower the interfacial tension between matrix of lipid and aqueous media [30,64].

PDI is a very important variable in nano systems because its value directly shows the homogeneity and uniformity of particles in the nano formulation. If the value of PDI is less than 0.3, the nano system is considered as uniform and stable [65,66]. There was an inverse relation between surfactant concentration and PDI, as by increasing the concentration of surfactant, there was significant decrease in PDI. On the contrary, the main reason for increase in PDI was the formation of non-uniform NT due to high lipid concentration [63]. The nonsignificant effect of drug content on PDI could be because of the effective method selection for the preparation of NT, leading to production of stable product.

Stability of formulation mainly depends on the charge of nanoparticles. ZP values in range between −25 and +25 is considered as indicator of good stability [67]. Herein, the negatively charged PL90G molecules creates vesicles with binary lipid layers are thought to be reason for negative ZP of NTZ-QUR-NT [46]. Moreover, considering Tween® 80 increases surface activity, the result implied that, it did adsorb on the particle surfaces by displacing the endogenous surface-active molecules [68]. As it is a non-ionic surfactant, a decrease in net charge at the particle surface was attributed to increase in its adsorption on the surface of NTZ-QUR-NT [69].

The effectiveness of nanoparticles mainly depends on entrapment of drug, thus it is considered a very important parameter for optimization of nanoparticles [70]. In NTZ-QUR-NT the entrapment of both QUR and NTZ were found to be significantly increased by increasing lipid concentration. Actually, increase in lipid concentration enhances the solubilization of lipophilic drugs and create more spaces for more drug molecules resulting in increased entrapment [71]. The %EE of both drugs were reduced when the surfactant concentration increased. Because, surfactant aligns in the lipid bilayer and lipophilic drugs competes for space, which leads to reduction in entrapment [72]. The optimized formulation has suitable particle size, PDI, ZP and entrapment efficiency.

Moreover, suitable morphology, and uniform particle distribution was evident from the TEM analysis confirming the zetasizer reports. Deformability is a unique feature of transferosomes which makes it possible for them to pass through the tight junctions of skin with ease. It is reported earlier that Tween® 80, a non-ionic surfactant serves as an edge activator, and may be responsible for the high deformability index value. Additionally, because of its long, light, and highly malleable carbon chain, it has a high degree of deformability [73]. FTIR spectra of PM and NTZ-QUR-NT indicated all the specific peaks of NTZ, QUR and PL90G which clearly showed the compatibility of drugs and excipients in formulation.

NTZ-QUR-NTG was homogeneous, having optimal pH, spreadability, and gelling properties. In addition, the final product followed non-newtonian flow which is desirable for topical application and gels that display it are referred as shear thinning gels [74,75]. Moreover, the final formulation was found stable for at least 6 months in respect of all the testing parameters, which could be attributed to the optimal properties, suitable technique of preparation and nanotechnology-based drug delivery [76].

Initially, the burst release of both drug from NTZ-QUR-NT was due to un-entrapped drug or desorption of drugs from the surface of the vesicles. Later, the release of drug was slowed down, indicating a sustained release behavior [77,78]. The sustained release of both drugs from NTZ-QUR-NT and NTZ-QUR-NTG would prevent frequent dosing [45]. The release of NTZ and QUR was significantly lower (*p* < 0.05) from NTZ-QUR-NTG as compared to dispersions. This is because in case of NTG the drugs pass through two barriers: first, from the NT and then through the chitosan gel [29,45]. Release pattern of QUR from its dispersion was different from NTZ dispersion due to acidic nature of QUR. This nature accounts for 100 % release of the drug in just 3 h. QUR release from QUR dispersion was more at pH 7.4 in comparison to pH 5.5 due to its acidic nature as it is more solubilized in basic media. But, there was no significant difference in release of drugs from NT and NTG at both pH [79,80]. The value of diffusion exponent (n) is considered key factor in release kinetic study, which is used to confirm that whether the release of drugs from drug delivery system is fickian or non-fickian diffusion [81]. Herein, the values of n advocated that QUR and NTZ followed fickian diffusion from NT and non-fickian from NTG for their drug release.

The flexibility and trans epidermal hydration gradient of NT make them squeeze across SC, but, transcutaneous hydration gradient enabled them to remain entrapped in dermis which is desired in case of cutaneous *Leishmaniasis* treatment [82,83]. The ex-vivo permeation study quantifies the amount of drug available to the body, offers vital information about the NTZ-QUR-NT behavior in in-vivo setting. The key mechanism of NT skin permeation into the SC and deeper skin layer is *trans*-epidermal osmotic gradient. The NTZ-QUR-NT experience no more water influx gradient when they reach the moist viable epidermis which retards their further movement [84]. This function is very important for enhancing skin retention of drugs at the infection site for a longer period of time and aids in minimizing the systemic toxicity [72]. In the current investigation, it was discovered that drug retention in deeper skin layers was higher than drug permeation, which is helpful for the topical therapy of CL since more dermal retention is needed than permeability.

The integrity and fluidity of skin layers lipids, especially which are present in the SC are altered by various polymeric nanoparticles as they move through the skin [85]. Thus, skin FTIR analysis was conducted to examine any effect of NT on lipids in the skin layers. The FTIR spectra demonstrated that NTZ-QUR-NT have no detrimental effects on skin structures and it was determined that their penetration through upper skin layers was because of their capability to transform shape [29]. Any dosage form intended for topical application to the skin should be non-toxic and non-irritating. The NTZ-QUR-NTG offers a better safety profile as shown by this investigation. The results of the skin irritation test were also supported by a histopathology analysis which confirms that NTZ-QUR-NTG didn't damage any skin tissues and no sign of inflammation were observed.

The key obstacle to effective treatment in CL is the inability of anti-leishmanial medications to enter macrophages. Since, the parasites reside inside the macrophages, the effectiveness of anti-leishmanial formulations mainly relies on how well it targets and accumulates there [55,86]. So, targeting of the infected macrophages was one of the main goals of the current study which was indicated using macrophage uptake and quantitative macrophage uptake studies. It was clear from the results that NTZ-QUR dispersion was distributed rather than particularly internalized by macrophages. It was expected that NTZ and QUR anti-leishmanial activity and potential might be greatly improved when entrapped within NT because of the suitable vesicle properties and good localization of NT in the infected macrophages [87]. Furthermore, internalization of drug-loaded NT in macrophages act like a secondary drug depot and improves drug availability to parasites in deeper skin layers [88].

Anti-leishmanial activity of NTZ and QUR is reported in many previous studies [[89], [90], [91]]. Anti-leishmanial activity of NTZ and QUR was significantly improved after encapsulation in NT. NTZ-QUR-NT were found highly effective mainly because of their nano range size, ultra-deformable nature and high macrophage internalization [26,92]. Moreover, negative charge on NT may stimulate the targeting due to their ligand binding ability with scavenger receptors of macrophages. An approach similar to that of annexins, the proteins of hydrophilic nature that reversibly bond with phospholipids negative charge and act as bridge in the process of vesicle fusion, may be used by negatively charged NT to target the parasite-phorous vacuoles inside macrophages [93]. The reduction in IC_50_ value of combination drugs may be due to the synergistic effect of both drugs which was confirmed by calculating the CI value [94]. The in-vivo antileishmanial data exhibited a significantly different reduction in the lesion size of the *Leishmania* after treatment with NTZ-QUR-NTG, which could be because of the ability of the NTZ-QUR-NTG to keep the formulation at the site of application and help the NT to cross the skin barriers and macrophage uptake, thereby releasing the co-loaded drug for efficient antileishmanial effects [24,45].

## 5 Conclusions

NTZ-QUR-NT were successfully developed, optimized and loaded into a chitosan-based gel for topical application. The optimized formulation had particles in nano-size range, adequate drug entrapment and good deformability. No physical and chemical interactions were found between components of NTZ-QUR-NT. Both the drugs showed sustained release from NTZ-QUR-NT and NTZ-QUR-NTG although the drug release from NTG was significantly different. Comparatively large amount of the drugs was observed to be retained in the deeper skin layers after application of the formulation which indicates the anti-leishmanial effectiveness of system. In-vivo skin irritation and histopathological findings didn't show any topical irritation associated with NTZ-QUR-NTG. Moreover, macrophage uptake analysis demonstrated that NTZ-QUR-NT were able to enhance macrophage internalization of both the drugs by passive targeting. Upon assessment in terms of anti-leishmanial potential, cell viability and toxicity the NTZ-QUR-NT system was found superior to NTZ-QUR dispersion. Overall, it was concluded that the NTZ-QUR-NTG had the ability to passively target the macrophages residing in the dermal layer with improved anti-leishmanial effect.

## Funding

This research work was funded by Higher Education Commission (HEC) of Pakistan through its grant No: 6171/Federal/NRPU R&D/HEC/2016 and grant No: 20-14604/NRPU/R&D/HEC/2021.

## Data availability statement

Data related to this article is presented in the form of results, tables, figures and supplementary material referenced in the article.

## CRediT authorship contribution statement

**Sidra Bashir:** Conceptualization, Investigation, Methodology, Software, Writing – original draft. **Kanwal Shabbir:** Data curation, Formal analysis, Investigation, Methodology, Writing – original draft. **Fakhar ud Din:** Conceptualization, Funding acquisition, Project administration, Supervision, Writing – review & editing. **Saif Ullah Khan:** Data curation, Formal analysis, Resources, Validation, Writing – review & editing. **Zakir Ali:** Data curation, Formal analysis, Methodology, Resources, Software, Visualization. **Barkat Ali:** Formal analysis, Resources, Validation, Visualization, Writing – original draft. **Dong Wuk Kim:** Conceptualization, Resources, Validation, Visualization, Writing – review & editing. **Gul Majid Khan:** Conceptualization, Funding acquisition, Project administration, Supervision.

## Declaration of competing interest

The authors declare that they have no known competing financial interests or personal relationships that could have appeared to influence the work reported in this paper.
