# Supplementary material for: Nitazoxanide and quercetin co-loaded nanotransfersomal gel for topical treatment of cutaneous leishmaniasis with macrophage targeting and enhanced anti-leishmanial effect
Source: Heliyon. 2023 Nov 2;9(11):e21939. doi: 10.1016/j.heliyon.2023.e21939 (PMC10661431; doi:10.1016/j.heliyon.2023.e21939)
Supplement: Multimedia component 1 [file mmc1.docx]

**Nitazoxanide and Quercetin Co-loaded Nanotransfersomal Gel for Topical Treatment of Cutaneous Leishmaniasis with Macrophage Targeting and Enhanced Anti-leishmanial Effect**

Supplementary Document

**Figure S1:** Preparation of NTZ-QUR-NT via thin film hydration. Figure is designed via Biorender


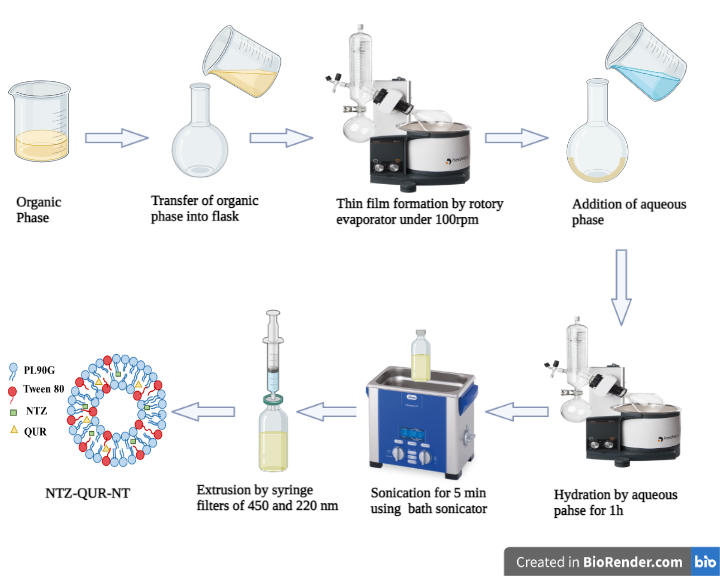


**Figure S2:** Method of quantitative macrophage uptake analysis


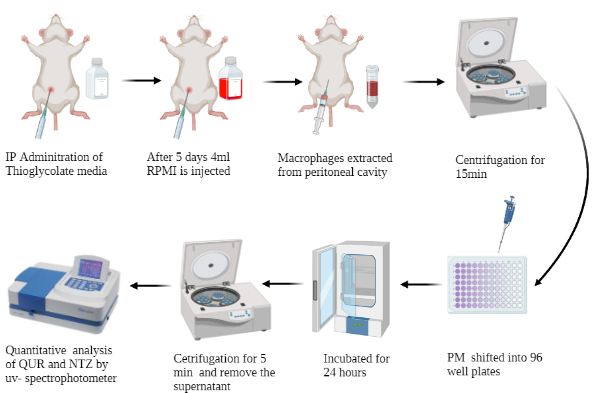


**Figure S3:** Evaluation of effect of shear rate on viscosity of NTZ-QUR-NTG


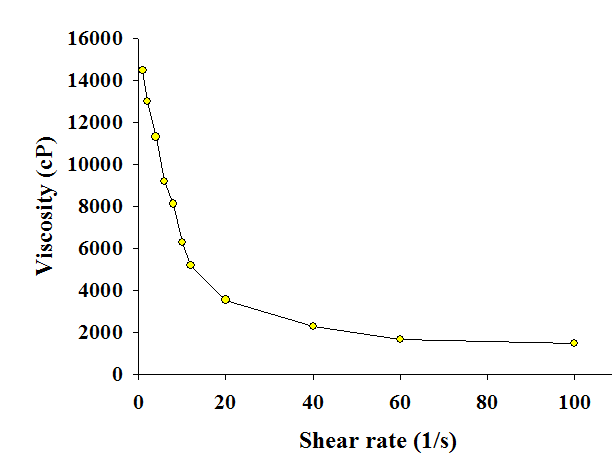


**Figure S4:** Variation of observed values from predicted values of Korsmeyer-Peppas model for NTZ-QUR-NT **(A)** NTZ; **(B)** QUR and Higuchi model for NTZ-QUR-NTG **(C)** NTZ; **(D)** QUR


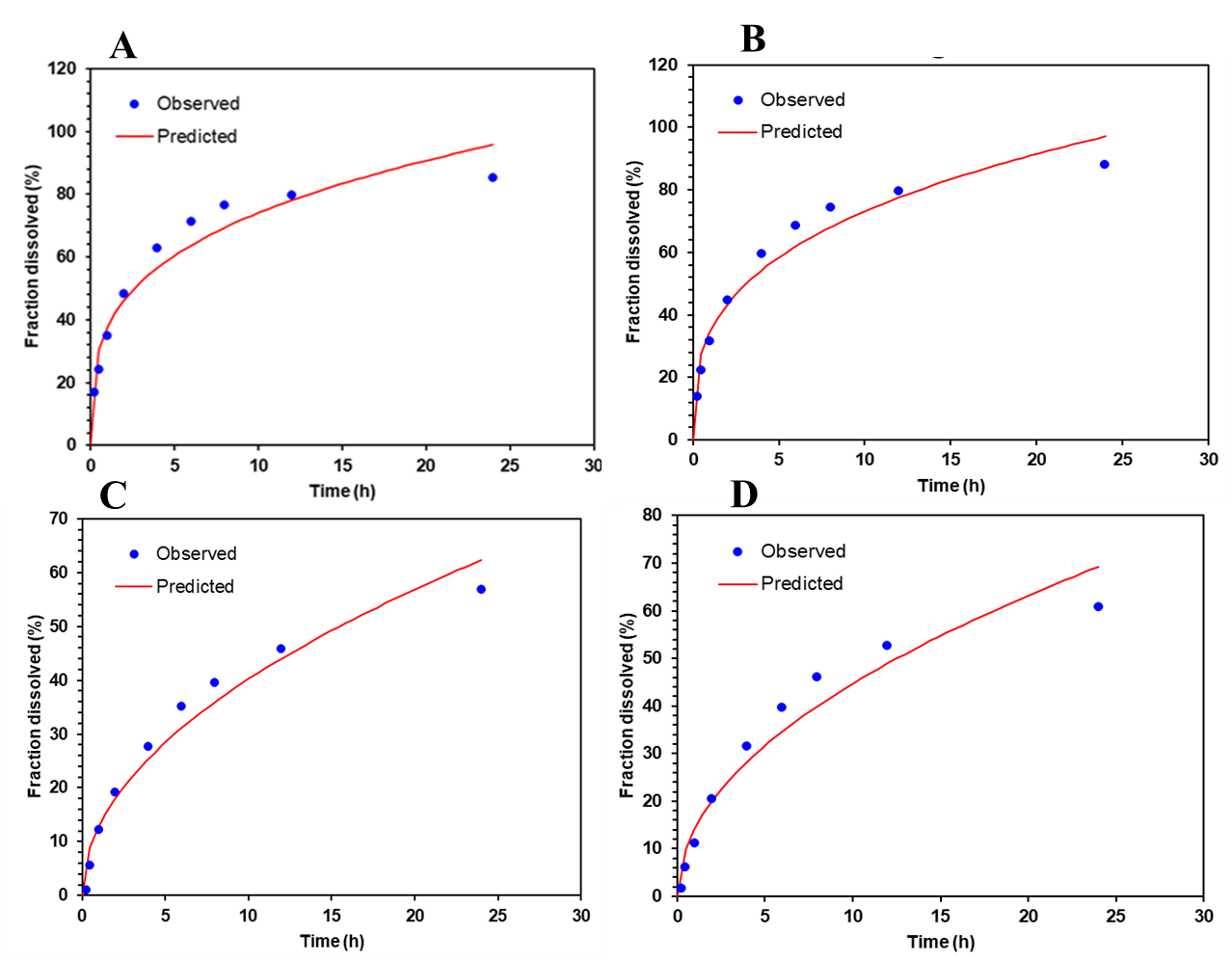


**Table S1:** Regression analysis of Box-Behnken design for all responses

| **Responses** | **R^2^** | **Predicted R^2^** | **Adjusted R^2^** | **Adequate precision** |
| --- | --- | --- | --- | --- |
| Particle size (nm) | 0.8952 | 0.8667 | 0.7762 | 18.2986 |
| PDI | 0.8587 | 0.8201 | 0.7010 | 15.5024 |
| ZP (mV) | 0.8469 | 0.8052 | 0.6815 | 13.3087 |
| %EE of QUR | 0.9198 | 0.8979 | 0.8286 | 20.1710 |
| %EE of NTZ | 0.9474 | 0.9330 | 0.9070 | 26.9197 |

**Table S2:** Characterization of the NTZ-QUR-NTG

| **Parameters** | **NTZ-QUR-NTG** |
| --- | --- |
| Physical Appearance | Yellowish opaque |
| Homogeneity | Uniform |
| Flow behaviour | Non-Newtonian flow |
| Drug content of NTZ | 98.45 ± 1.17 |
| Drug content of QUR | 97.86 ± 1.39 |
| pH | 5.8 ± 1.56 |
| Spreadability | 310.50 ± 3.5% |

**Table: S3.** Draize scoring data for skin irritation test.

| **Groups** | **Erythema** | | | | **Edema** | | | | **PDII** |
| --- | --- | --- | --- | --- | --- | --- | --- | --- | --- |
|  | 1 hr | 24 hr | 48 hr | 72 hr | 1 hr | 24 hr | 48 hr | 72 hr |  |
| **Normal** | 0 | 0 | 0 | 0 | 0 | 0 | 0 | 0 | 0 |
| **Formalin treated (0.8%)** | 3 | 2 | 2 | 2 | 1 | 2 | 2 | 2 | 2 |
| **NTZ-QUR-NTG treated** | 0 | 0 | 0 | 0 | 0 | 0 | 0 | 0 | 0 |

***Note:*** *PDII = Primary Dermal Irritation Index*
